# cFos Mediates cAMP-Dependent Generation of ROS and Rescue of Maturation Program in Retinoid-Resistant Acute Promyelocytic Leukemia Cell Line NB4-LR1

**DOI:** 10.1371/journal.pone.0050408

**Published:** 2012-11-28

**Authors:** Jean-Luc Carrier, Pasha Javadi, Emilie Bourrier, Céline Camus, Evelyne Ségal-Bendirdjian, Aïda Karniguian

**Affiliations:** 1 INSERM UMR-S 1007, Paris, France; 2 Université Paris Descartes, Sorbonne Paris Cité, Paris, France; Institut Jacques Monod, France

## Abstract

A determining role has been assigned to cAMP in the signaling pathways that relieve resistance to anti-leukemia differentiation therapy. However, the underlying mechanisms have not been elucidated yet. Here, we identify cFos as a critical cAMP effector, able to regulate the re-expression and splicing of epigenetically silenced genes associated with maturation (*CD44)* in retinoid-resistant NB4-LR1 leukemia cells. Furthermore, using RNA interference approach, we show that cFos mediates cAMP-induced ROS generation, a critical mediator of neutrophil maturation, and *in fine* differentiation. This study highlights some of the mechanisms by which cAMP acts to overcome resistance, and reveals a new alternative cFos-dependent pathway which, though nonexistent in retinoid-sensitive NB4 cells, is essential to rescue the maturation program of resistant cells.

## Introduction

Acute promyelocytic leukemia (APL) cells are characterized by the t(12;17)(q22;q12) chromosomal translocation, leading to a blockade of their differentiation into mature granulocytic cells. Although APL is a rather rare disease, it constitutes an invaluable model for the study of cancer biology and the development of new therapeutic strategies based on differentiation. All-*trans* retinoic acid (ATRA) is well known to induce the maturation of APL cells into neutrophils [1]. Even though this agent is successfully used in therapy protocols, resistance to ATRA often develops, and approaches to avoid or reverse drug resistance are under intensive investigation.

Studies performed on the well-established NB4-LR1 cell line, derived from an ATRA-resistant APL patient, have highlighted the importance of signaling synergies to overcome resistance [2], [3], [4], [5]. In particular, a determining role has been assigned to cAMP. Indeed, an analogue of cAMP (8-CPT-cAMP), in association with ATRA, proved able to reverse resistance and trigger terminal differentiation of the resistant APL NB4-LR1 cell line [4], [6]. Moreover, theophylline, a phosphodiesterase inhibitor known to stabilize intracellular cAMP levels, has restored normal hematopoiesis in an APL patient resistant to combined ATRA/As_2_O_3_ therapy [7]. The molecular mechanisms by which cAMP acts to normalize the phenotype of resistant leukemia cells are still poorly understood. Besides the already known mutations in the PML-RAR fusion gene [8], [9], our recent studies have revealed the existence of aberrant epigenetic events in ATRA-resistant NB4-LR1 cells, responsible for the downregulation of genes associated with differentiation [10]. This is the case for the *CD44* gene, encoding for a well-known receptor implicated in the maturation of myeloid cells. Repression of *CD44* is due to an aberrant methylation of its promoter, associated with deacetylation of histone H3. We have demonstrated that cAMP induces post-translational modifications (Ser10-phosphorylation/Lys14-acetylation) of histone H3 in bulk chromatin, known to favor the transcriptional activity of immediate early genes [11], [12], [13], as well as the expression and the dual Ser63/Ser73 phosphorylation of transcription factor cJun. The latter is further recruited concomitantly with chromatin remodeling factors to the AP-1 site of the *CD44* promoter, leading to the transcriptional re-expression of *CD44* and the restoration of a functional receptor at the surface of NB4-LR1 cells [10]. These results strongly suggest that cAMP may reverse the silencing of genes through chromatin remodeling and transcription factor activation.

**Figure 1:**
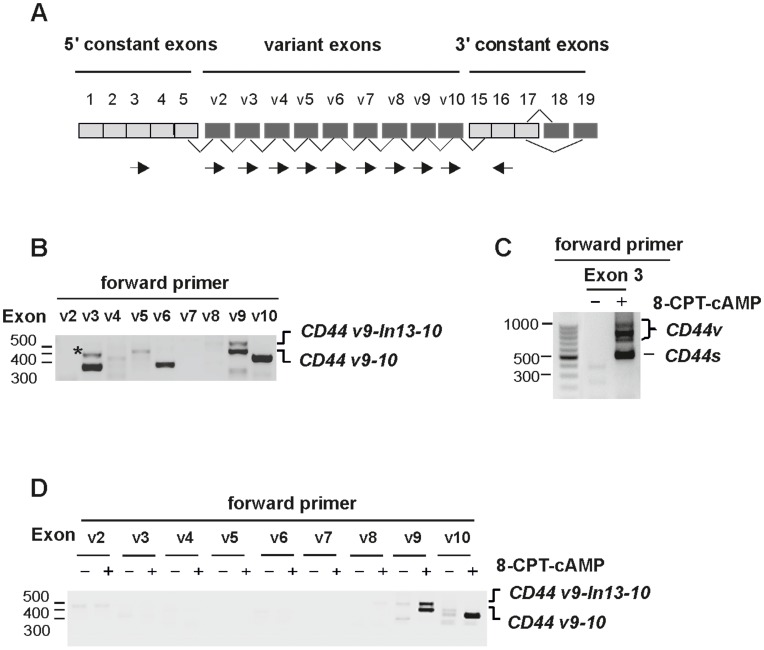
cAMP regulates alternative splicing of CD44 in ATRA-resistant NB4-LR1 cells. (**A**) *Schematic representation of the genomic structure of CD44 gene.* Constitutively (grey) or alternatively (dark grey) spliced exons, and position of exon-specific forward and reverse (arrows) primers used in RT-PCR are shown. (**B**) *Splicing pattern of CD44 in NB4 cells*. *CD44* sequence was amplified by RT-PCR using exon specific forward primers (v2 to v10) in combination with a constant exon 16 specific reverse primer. PCR products were analyzed on agarose gel stained by ethidium bromide, cloned and sequenced. NB4 cells express variant exons v3 and v6 as a single exon, and exon v9 in combination with v10 (*CD44*v9-10). The upper band in the “v9” lane corresponds to a new *CD44* variant with a 127 bp intronic sequence inclusion (*CD44*v9-In13-10). The expression of single exon v10 is shown in the *“*v10” lane. The asterisk corresponds to a non specific band (“v3” lane). (**C**, **D**) *Splicing pattern of CD44 in resistant NB4-LR1 cells.* Cells were mocked (−) or treated (+) with 8-CPT-cAMP (150 µM). *(C) CD44* sequence was first amplified by RT-PCR using primers spanning the alternatively splice insertion site (forward primer in constant exon 3 and reverse primer in constant exon 16). Both *CD44* standard (*CD44*s) and spliced isoforms (*CD44*v) were induced by 8-CPT-cAMP. (D) *CD44* sequence was further amplified using variant exon specific forward primers (v2 to v10) and a reverse primer in the constant exon 16. Only the expression of *CD44*v9-10 and *CD44*v9-In13-10 variants is induced by 8-CPT-cAMP. The expression of exon v10 is also shown (*“*v10” lane).

The present study confirms this hypothesis and reveals that cAMP effects are mediated through early induction of cFos protein. The latter is found to be a critical upstream factor involved in the re-expression of the silenced *CD44* gene. A novel CD44 variant associated with APL is more particularly identified. cFos transcription factor is also shown to act as a fine cAMP-signaling effector, capable to rescue the maturation program of leukemia cells and relieve their retinoid resistance.

## Materials and Methods

### Cell Lines and Treatments

NB4, a well-established permanent cell line [1], and NB4-LR1, a NB4-derived retinoid-resistant cell line [14], [15], were cultured in RPMI 1640 medium supplemented with 2 mM L-glutamine, 100 units/ml penicillin, 0.1 mg/ml streptomycin and 10% fetal calf serum (Invitrogen). Cells were exposed to 1 µM all-*trans* retinoic acid (ATRA), or to 150 µM 8- (4- Chlorophenylthio) adenosine- 3′, 5′- cyclic monophosphate (8-CPT-cAMP) in combination or not with ATRA.

**Figure 2:**
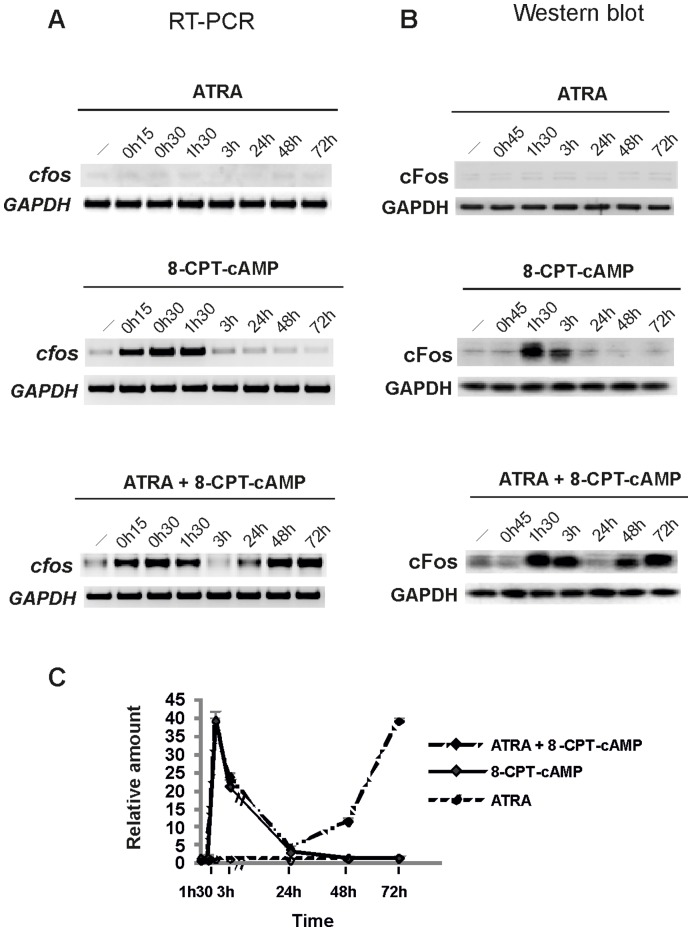
Time course of cFos expression in response to ATRA and 8-CPT-cAMP in NB4-LR1 cells at transcriptional and protein levels. (**A**) *Representative RT-PCR analysis* of c*fos* mRNAs from NB4-LR1 cells, mocked (−) or treated with ATRA (10^−6 ^M), 8-CPT-cAMP (150 µM) or in combination, as indicated. (**B**) *Western blot analysis of cFos expression*. Proteins from total lysates of NB4-LR1 cells, mocked (−) or treated with ATRA and/or cAMP as indicated, were resolved on 10% SDS–polyacrylamide gel electrophoresis (PAGE) and processed for immunoblotting with anti-cFos antibody (Santa Cruz). *Glyceraldehyde-3-phosphate dehydrogenase* (GAPDH) served as a loading control. (**C**) *Quantitative expression of cFos protein relative to control*. Western blot signals were scanned and cFos protein expression levels were quantitated by using FluorChem 8800 Imager. The mean value (± s.d) from at least four independent experiments is indicated. No induction is observed with ATRA, whereas cFos is transiently induced in response to 8-CPT-cAMP. A biphasic expression pattern is observed in response to 8-CPT-cAMP and ATRA, which cooperate to overcome resistance.

### RT-PCR Analysis

Total RNAs purified from NB4 and NB4-LR1 cells were isolated using the RNeasy Kit (Qiagen), and reversed transcribed using 200 U MMLV reverse transcriptase enzyme (Promega). PCR amplification of *CD44* transcripts was performed with specific primers, as indicated in Table S1, for 30 cycles of denaturation at 94°C for 30 sec, annealing at 52°C for 60 sec, and extension at 72°C for 75 sec, using 2.5 units of DyNAzyme DNA polymerase (Thermo Fisher Scientific). For amplification of *cfos* and *c-jun* transcripts, annealing was performed at 56°C, and the following primers were used: *cfos* forward 5′-CCAACTTCATTCCCACGTC-3′ and reverse 5′-CTCCCTCCTCCGGTTGC-3′; *c-jun* forward 5′-GACTGCAAAGATGGAAACGA-3′ and reverse 5′-GTTGCTGGACTGGATTATCA-3′. Primers specific to the glyceraldehyde-3-phosphate dehydrogenase gene were used for loading controls: 5′-CTCAGACACCATGGGGAAGGTGA-3′ and 5′-ATGATCTTGAGGCTGTTGTCATA-3′.

**Figure 3:**
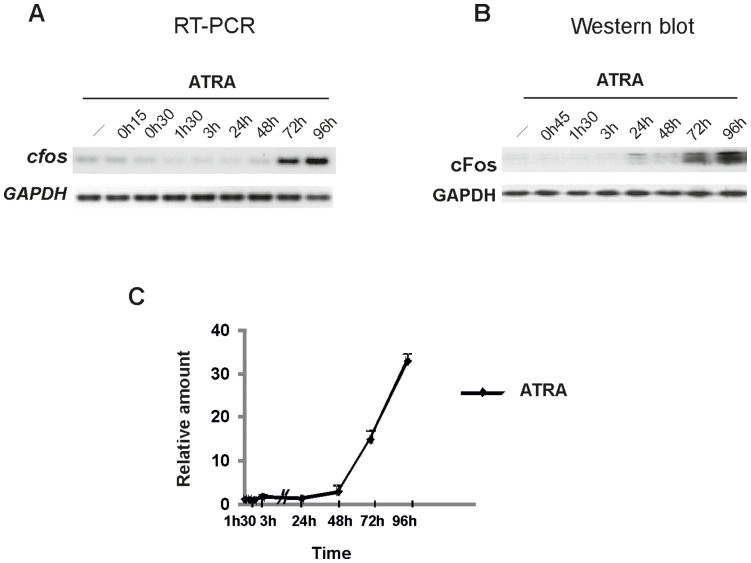
Time course of cFos expression in response to ATRA in NB4 cells at transcriptional and protein levels. (**A**) *Representative RT-PCR analysis* of c*fos* mRNAs from NB4 cells, mocked (−) or treated with ATRA (10^−6 ^M), as indicated. (**B**) *Western blot analysis of cFos expression*. Proteins from total lysates of NB4 cells, mocked (−) or treated with ATRA, were resolved on 10% SDS–polyacrylamide gel electrophoresis (PAGE) and processed for immunoblotting with anti-cFos antibody (Santa Cruz). *Glyceraldehyde-3-phosphate dehydrogenase* (GAPDH) served as a loading control. (**C**) *Quantitative expression of cFos protein relative to control*. Western blot signals were scanned and cFos protein expression levels were quantitated by using FluorChem 8800 Imager. The mean value (± s.d) from at least four independent experiments is indicated. cFos is induced as a terminal differentiation factor in NB4 cells in response to ATRA treatment (after 72 h).

### Western Blot Analysis

NB4 or NB4-LR1 cells, treated as indicated, were resolved by 10% SDS-PAGE and transferred onto 0.2 µm nitrocellulose membranes (BA85, Schleicher & Schuell). The membranes were then probed with a cFos specific antibody (H-125, Santa Cruz) and the immuno-reactive bands were revealed by chemiluminescence detection (ECL, Amersham). Immunoblots were further scanned to quantify band intensities using FluorChem analysis software.

**Figure 4:**
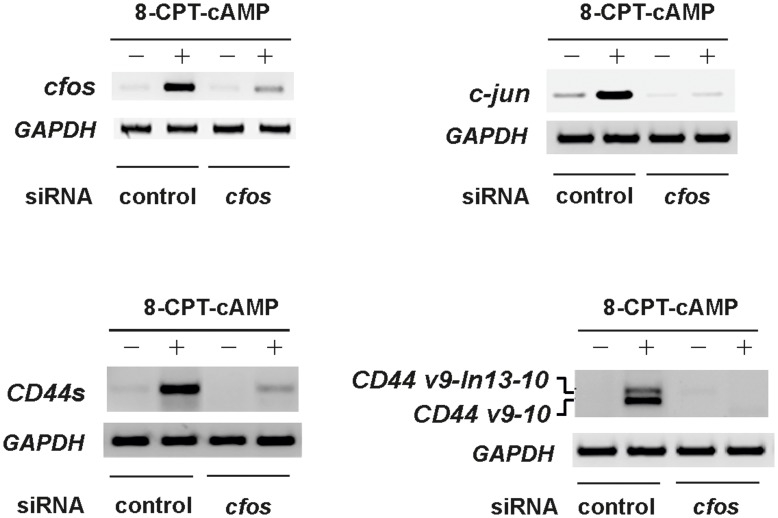
*cfos* silencing with siRNAs inhibits the cAMP-mediated expression of *c-jun and CD44*. (**A**) *cfos siRNA silencing efficiency* assessed by RT-PCR analysis from NB4-LR1 cells treated with 8-CPT-cAMP (150 µM). Specific pool siRNAs reduce *cfos* mRNA levels by at least 80%. (**B–D**) *Silencing of cfos results in inhibition of c-jun (B), CD44 standard form (CD44s) (C) and CD44 splice variant (CD44v) (D) expression,* as assessed by RT-PCR analysis using specific primers.

### RNA Interference

A set of four Dharmacon ON-TARGET*plus* SMARTpool siRNAs (Thermo Fisher Scientific) specifically targeting human *cfos* were delivered to cells by electroporation. Dharmacon ON-TARGET*plus siCONTROL* Non-Targeting siRNAs were used as negative control (control siRNA). NB4-LR1 cells were washed and re-suspended in Opti-MEM medium (Invitrogen) with 50 nM of siRNAs at a concentration of 4×10^6/^cuvette. Electroporation was performed with a single 150 µs pulse of 260 V (Gene Pulser II, Biorad). Cells were then mock- or 8-CPT-cAMP-treated, in combination or not with ATRA, and further analyzed for functional studies, as indicated.

**Figure 5:**
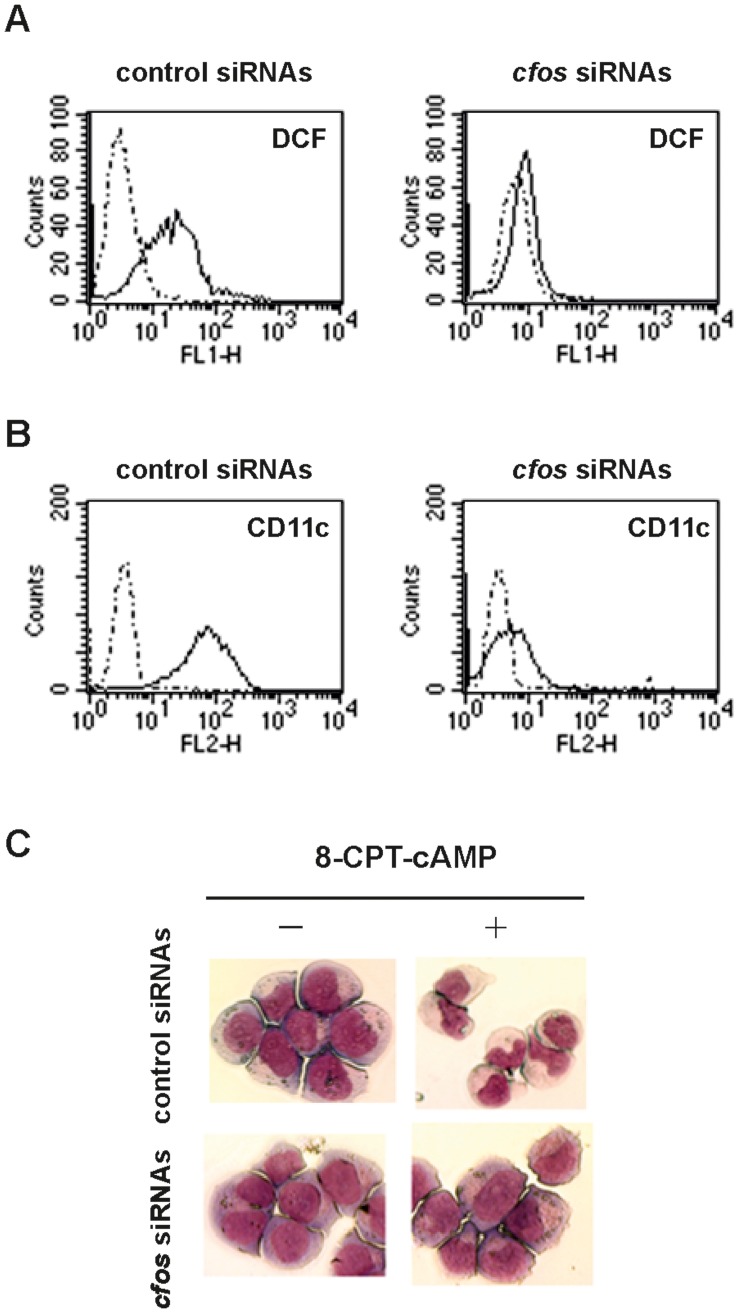
*cfos* silencing with siRNAs reduces ROS generation, inhibits cell surface expression of CD11c and affects terminal differentiation of NB4-LR1 cells induced by cAMP/ATRA cooperation. (**A**) *Silencing of cfos results in downregulation of ROS generation.* FACS analyses of intracellular reactive oxygen species (ROS) generation in mock- (dotted line) or 8-CPT-cAMP/ATRA-treated (full line) NB4-LR1 cells, evaluated by the measurement of 2, 7-dihydrodichlorofluorescein (DCF) fluorescence, are shown. Representative histograms of FITC staining represent an acquisition of 10^4^ events. (**B**) *Silencing of cfos inhibits the cell surface expression of CD11c.* FACS analyses of CD11c expression on the surface of mock- (dotted line) or 8-CPT-cAMP/ATRA-treated (full line) NB4-LR1 cells are shown. Fluorescence intensity was evaluated by using a PE-conjugated anti-CD11c antibody. Representative histograms of FITC staining represent an acquisition of 10^4^ events. (**C**) *Silencing of cfos affects terminal differentiation of NB4-LR1 cells in response to 8-CPT-cAMP/ATRA cooperation.* Morphology of mock- (left) or 8-CPT-cAMP/ATRA-treated (right) NB4-LR1 cells, transfected with control (top) or *cfos* specific siRNAs (bottom) as indicated, is shown. Cells were stained with May-Grünwald-Giemsa solution. Images were acquired and processed using a Leica microscope (x63) and the QWin software.

### Detection of Reactive Oxygen Species (ROS) Formation

siRNA electroporated NB4-LR1 cells, treated or not with 8-CPT-cAMP and ATRA for 24 h, were loaded with 5-(and 6-)chloromethyl-2′,7′-dichlorodihydrofluorescein diacetate (CM-H2DCF-DA, Invitrogen) in the presence of 0.3 µM PMA, for 20 minutes at 37°C in the dark. The fluorescent reaction product (DCF) that was obtained was analyzed by flow cytometry using a FACS-Calibur instrument (Becton Dickinson Immunocytometry Systems) and CellQuest software.

### Cell Surface Expression of CD11c in NB4-LR1 Cells

siRNA electroporated NB4-LR1 cells, treated or not with 8-CPT-cAMP and ATRA, were incubated with a phycoerythrin-conjugated anti-CD11c antibody or the isotope-matched non-specific IgGs (BD Pharmingen) for 30 minutes at 4°C. Cells were then washed, fixed in 1% paraformaldehyde and analyzed by flow cytometry using a FACS-Calibur instrument (Becton Dickinson Immunocytometry Systems) and CellQuest software. Results were expressed as histograms of PE-staining and quantified as the relative mean fluorescence intensity.

### Morphological Analysis of NB4-LR1 Cells

siRNA electroporated NB4-LR1 cells were exposed to a combination of 8-CPT-cAMP/ATRA for 48 h. Cells were then centrifuged by cytospin techniques onto glass sides, stained in May-Grünwald-Giemsa solution, and analyzed for cell morphology using a 63X objective.

## Results and Discussion

To gain additional insight into the role of cAMP, we first pursued our investigations into the reactivation of the *CD44* gene. We show here that cAMP may also regulate alternative splicing, a process of primary importance to introduce an additional level of gene regulation. The human *CD44* gene, which contains nine variable exons (exon 6–14), can indeed be spliced into a large number of combinations (CD44 v2-v10) (Figure 1A). A complex pattern of *CD44* variant expression is generally observed in acute myeloid leukemia (AML) patients [16]. Nevertheless, three variant isoforms, v3, v6 and v9, are often expressed in the majority of AML cases, v6 being further associated with poor prognosis [17]. In the present study, RT-PCR analysis using exon-specific primers (Table S1), followed by sequencing, reveals that APL NB4 cells express *CD44* isoforms, either encoded by single-variant exons v3 and v6, or by exon v9 found to be associated with exon v10 (*CD44*v9-10) (Figure 1B). Furthermore, using a specific sense primer from v9, we also identified a novel form of *CD44* that contains a 127 bp intronic sequence located between exons v9 (exon 13) and v10 (exon14) (*CD44*v9-In13-10) (Figure 1B and Figure S1). Such abnormal retention of intronic sequences in CD44 transcripts has already been described in many cancer cells, in particular for introns 6 or 9 [16], [18], [19]. However, the retention of intron 13 is unusual. It has only been reported in breast tumors, embryonic tissues or myoblasts in mice [20], [21]. No evidence for the presence of this particular *CD44* variant in human cancer cells, in particular in AML or APL cells, has been shown to date.

In resistant NB4-LR1 cells lacking CD44 standard (CD44s), an isoform that comprises all constant exons (exons 1–5 and 16–19), no splice variants were detected. Upon cAMP treatment, these cells upregulated CD44s (Figure 1C), as previously shown [10]. Importantly, they also acquired a splicing pattern that resulted in the expression of the *CD44* v9-10 variant (Figure 1C, D). Of note, previous chromatin immunoprecipitation analysis (data not shown), using amplification primers encompassing exon v9, revealed cJun recruitment not only to the *CD44* promoter but also to this region, both containing a splicing enhancer and a potential AP-1 motif sequence. Interestingly, cAMP also induced the expression of the novel *CD44* splice variant with the inclusion of intron 13 (*CD44*v9-In13-10) (Figure 1D and Figure S1). This finding is in agreement with a previous paper reporting that the splicing of this particular intronic sequence, studied by using minigene construct systems, is under high specific control [22].

These observations prompted us to identify early effectors of cAMP able to act upstream of this cascade of events. Regulated by two important response elements, CRE and SRE, c*fos* is an early immediate gene responsive to cAMP. This widely-known transcription factor is generally implicated in cell proliferation and transformation. However, cFos has been reported to have a role in the end-stage maturation of hematopoietic cells [23], [24], [25], [26], including osteoclasts [27], [28], [29], as well as in the end-stage maturation of keratinocytes [30] or neuronal cells [31], [32]. We therefore investigated whether cFos played a role in reprogramming resistant APL cells towards differentiation in response to cAMP/ATRA cooperation.

We first studied its expression in NB4-LR1 cells treated with cAMP, ATRA or a combination of ATRA and cAMP, both at the transcriptional and protein levels. As previously reported, ATRA or cAMP signaling is not self-sufficient to provoke the maturation of retinoid-resistant cells, and a crosstalk between the two drugs is necessary to trigger differentiation [4], [5]. As shown in Figure 2A, whereas no effect of ATRA was seen in resistant NB4-LR1 cells, 8-CPT-cAMP treatment strongly increased *cfos* mRNA levels after 15 min. Western blotting analysis (Figure 2B) also showed a transient induction of cFos protein, with a peak at 90 min. In absence of maturation, both mRNA and protein levels rapidly returned to control values within 1h30 and 3 h, respectively. In association with ATRA, 8-CPT-cAMP induced a biphasic response in *cfos* mRNA and protein expression (Figure 2A, B), *i.e.* an early induction identical to that observed in NB4-LR1 cells treated with cAMP alone, followed by a second one, more sustained, which was associated with a terminal stage in the progression of promyelocytic cells to mature neutrophils (Figure 2C).

Of note, in retinoid-sensitive NB4 parental cells, which are responsive to ATRA alone, only a late induction of cFos is observed (Figure 3A–C), associated with the terminal differentiation of cells, as has been reported for osteoclasts or other terminally maturated cell types. ATRA was found incapable to mediate a fast induction of cFos, suggesting a differential effect of ATRA and cAMP/ATRA association on the transduction of maturation signals in NB4 and NB4-LR1 cells, respectively. These results raise the hypothesis that the early induction of cFos by cAMP may represent an important signal enabling resistant cells to differentiate.

A gene-silencing approach has then been considered to determine whether cAMP-induced cFos was implicated in relieving ATRA resistance. A pool of specific short interfering RNA (siRNA) was used, and their efficiency in knocking down c*fos* mRNAs was confirmed (Figure 4A). In a first set of experiments, we showed that suppressing cAMP-induced *cfos* by siRNAs greatly inhibited the expression of *c-jun (* Figure 4B), as well as those of *CD44*s and its splice v9-10 variant (Figure 4C, D).

We further assessed the implication of cFos in the transduction of differentiation signals. We first examined whether its silencing might affect the production of reactive oxygen species (ROS), considered as an important marker of neutrophil maturation. Extinction of cAMP-induced c*fos* significantly inhibited the generation of intracellular ROS (Figure 5A), as well as the expression of CD11c, a cell surface marker of NB4 maturation (Figure 5B), evaluated by fluorescent analysis. Consistently, c*fos* silencing also resulted in the inhibition of differentiation of resistant APL cells into neutrophils (Figure 5C). NB4-LR1 cells cultured for 2 ½ days with the combination of cAMP and ATRA presented typical morphological changes. They decreased their nuclear/cytoplasmic ratio and staining, developing condensed, lobulated nuclei, characteristic of cells undergoing granulocyte-like maturation (Figure 5C, top right). In contrast, c*fos* siRNA transfected resistant cells displayed the morphology of undifferentiated APL cells with large nuclei surrounded by a shell of more basophilic cytoplasm (Figure 5C, bottom right).

### Conclusion

In summary, this study reveals for the first time the existence of a novel inducible *CD44* splice variant in APL cells, highly regulated by cAMP. This work also provides new insights into molecular mechanisms by which cAMP acts to sensitize blasts that are resistant to differentiation. Early cAMP-induced cFos appears to be a critical reprogramming factor that, working upstream of a signaling cascade, is essential to relieve the transcriptional repression of *CD44* gene and of its splice variant. It further triggers the generation of ROS, a critical mediator of neutrophil maturation and *in fine* the terminal differentiation of resistant cells. Interestingly, early-induced cFos dependent pathway does not occur during ATRA-sensitive NB4 cell differentiation. This result strongly suggests that cAMP, able to regulate a complex set of cellular events, induces novel differentiation signals to restore maturation of resistant cells, rather than repairing defective pathways. Our work supports the existence of a cFos-dependent alternative pathway in NB4-LR1 cells, which deepens our understanding of maturation signals overcoming resistance, and may uncover novel therapeutic opportunities.

## Supporting Information

Figure S1 **Sequence of a new alternatively spliced** ***CD44*** **variant in APL cells (v9-In13-v10).** Amplifications of mRNAs from NB4 and cAMP-treated NB4-LR1 cells were performed using a forward primer in variant v9 (exon 13) and a reverse primer in constant exon 16. Sequencing of PCR products revealed the presence of an intronic sequence between exon v9 (exon 13) and exon v10 (exon 14), which corresponds to the first 127 bp of intron 13 (*CD44*, RefSeqGene: NG_008937.1). An in-frame stop codon (italic, ***) is generated in the intronic sequence, probably leading to a soluble form of *CD44, as* the *trans*membrane domain is carried by exon 17.
(PPTX)

Table S1 Primer sequences used for RT-PCR and expected amplicon sizes.
(PDF)
