# Supplementary material for: cFos Mediates cAMP-Dependent Generation of ROS and Rescue of Maturation Program in Retinoid-Resistant Acute Promyelocytic Leukemia Cell Line NB4-LR1
Source: PLoS One. 2012 Nov 28;7(11):e50408. doi: 10.1371/journal.pone.0050408 (PMC3508928; doi:10.1371/journal.pone.0050408)
Supplement: Table S1 — Primer sequences used for RT-PCR and expected amplicon sizes. (PDF) [file pone.0050408.s002.pdf]

Primer designation	Primer sequence 5'→3'	Expected amplicon size (bp)
**Exon 3** *forward*	TGGGTTCATAGAAGGGCATG	***Exon 3 → Exon 16***<br>***CD44s*** [1] : 565<br>***CD44v9*** [2] : 655<br>***CD44v10*** [2] : 769<br>***CD44v9-v10*** [3] : 859<br>***CD44v9-In13-v10*** [3] : 986
**v2** / **Exon 6** *forward*	GCTACAGCAACTGAGACAGC	***v2 → Exon 16***<br>**v2** [2] : 243
**v3** / **Exon 7** *forward*	ACGTCTTCAAATACCATCTC	***v3 → Exon 16***<br>**v3** [2] : 255
**v4** / **Exon 8** f*orward*	CCAGGACTGGACCCAGTGGAA	***v4 → Exon 16***<br>**v4** [2] : 205
**v5** / **Exon 9** *forward*	GTAGACAGAAATGGCACCAC	***v5 → Exon 16***<br>**v5** [2] : 246
**v6** / **Exon 10** *forward*	CAGGCAACTCCTAGTAGTAC	***v6 → Exon 16***<br>**v6** [2] : 258
**v7**/ **Exon 11** *forward*	CCAGCCATCCAATGCAAGGA	***v7 → Exon 16***<br>**v7** [2] : 248
**v8** / **Exon 12** *forward*	CGCTTCAGCCTACTGCAAATCC	***v8 → Exon 16***<br>**v8** [2] : 209
**v9** / **Exon 13** *forward*	GCTTCTCTACATCACATGAAGG	***v9 → Exon 16***<br>**v9** [2] : 203<br>**v9+v10** [3] : 407<br>**v9+in13+v10** [3] : 530
**v10**/ **Exon 14** *forward*	AGGAATGATGTCACAGGTGG	***v10 → Exon 16***<br>**V10** [2] : 335
**Exon 16** ***reverse***	ATTTGGGGTGTCCTTATAGG	-

[1] No inclusion of variant exon
[2] Inclusion of single variant exon
[3] Inclusion of multiple variant exons
